# Supplementary material for: Single-molecule variation in telomeric sequence and structure across humans
Source: bioRxiv. 2026 May 5:2026.05.01.722200. Preprint. [Version 1] doi: 10.64898/2026.05.01.722200 (PMC13174434; doi:10.64898/2026.05.01.722200)

Supplemental Figure 1

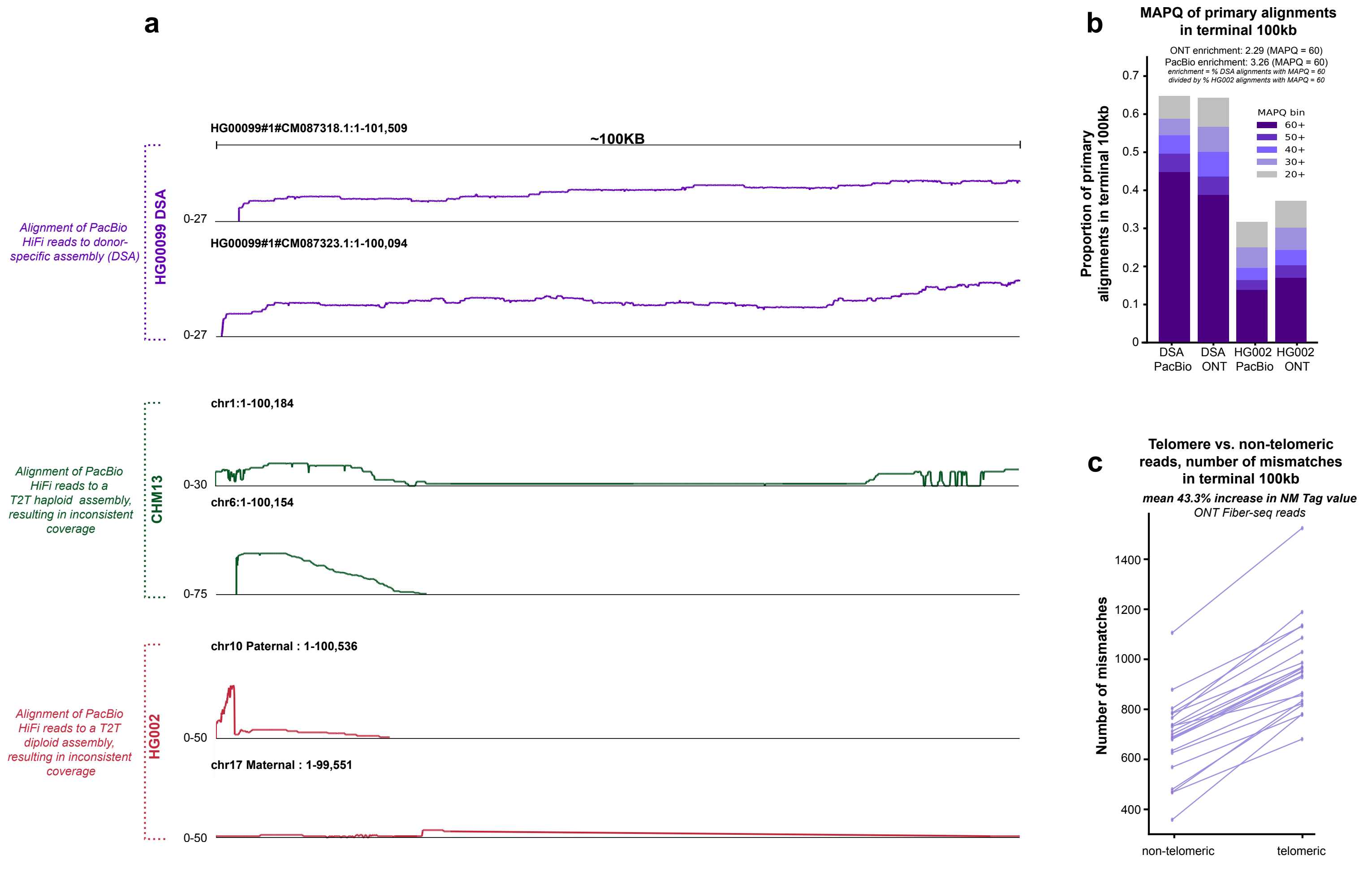

Supplemental Figure 2

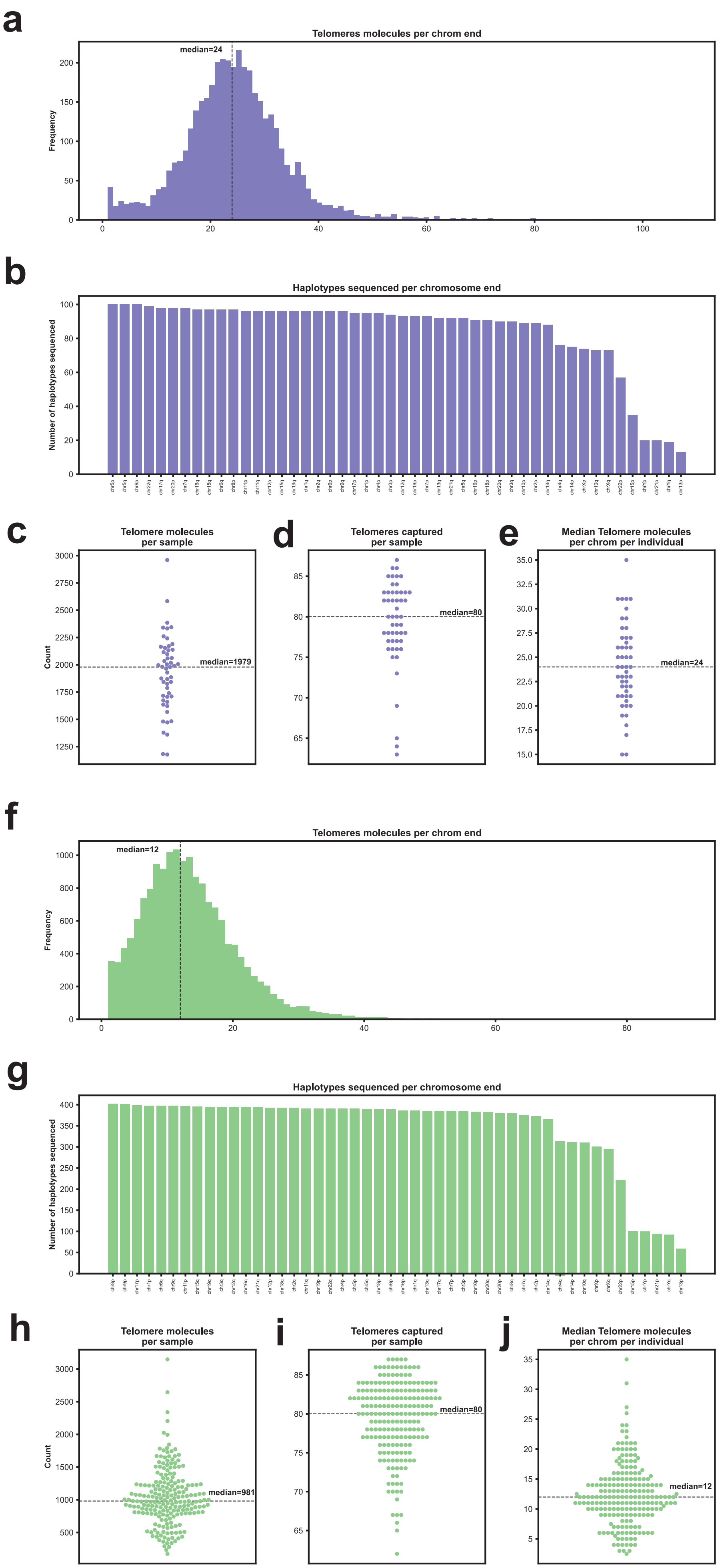

Supplemental Figure 3

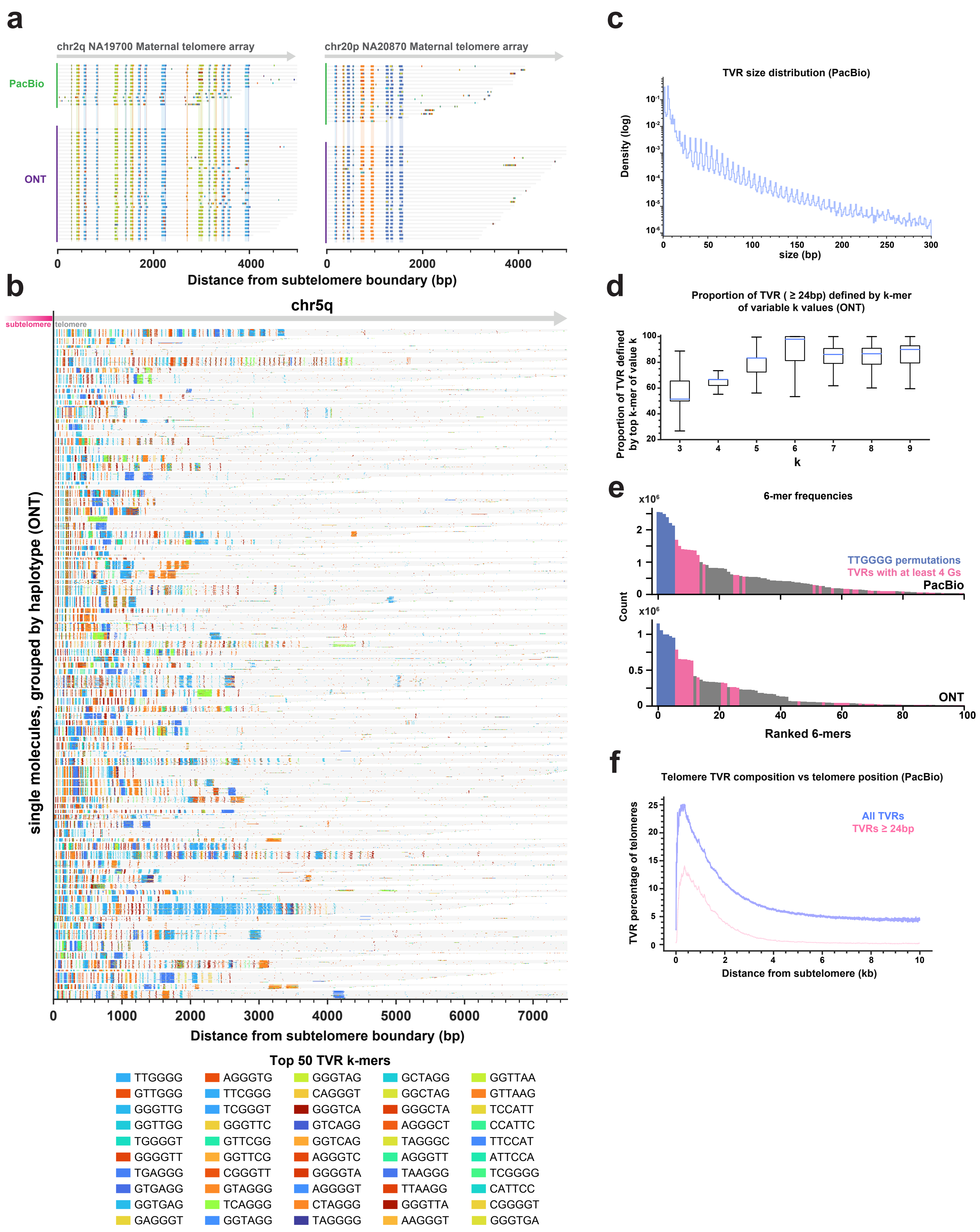

Supplemental Figure 4

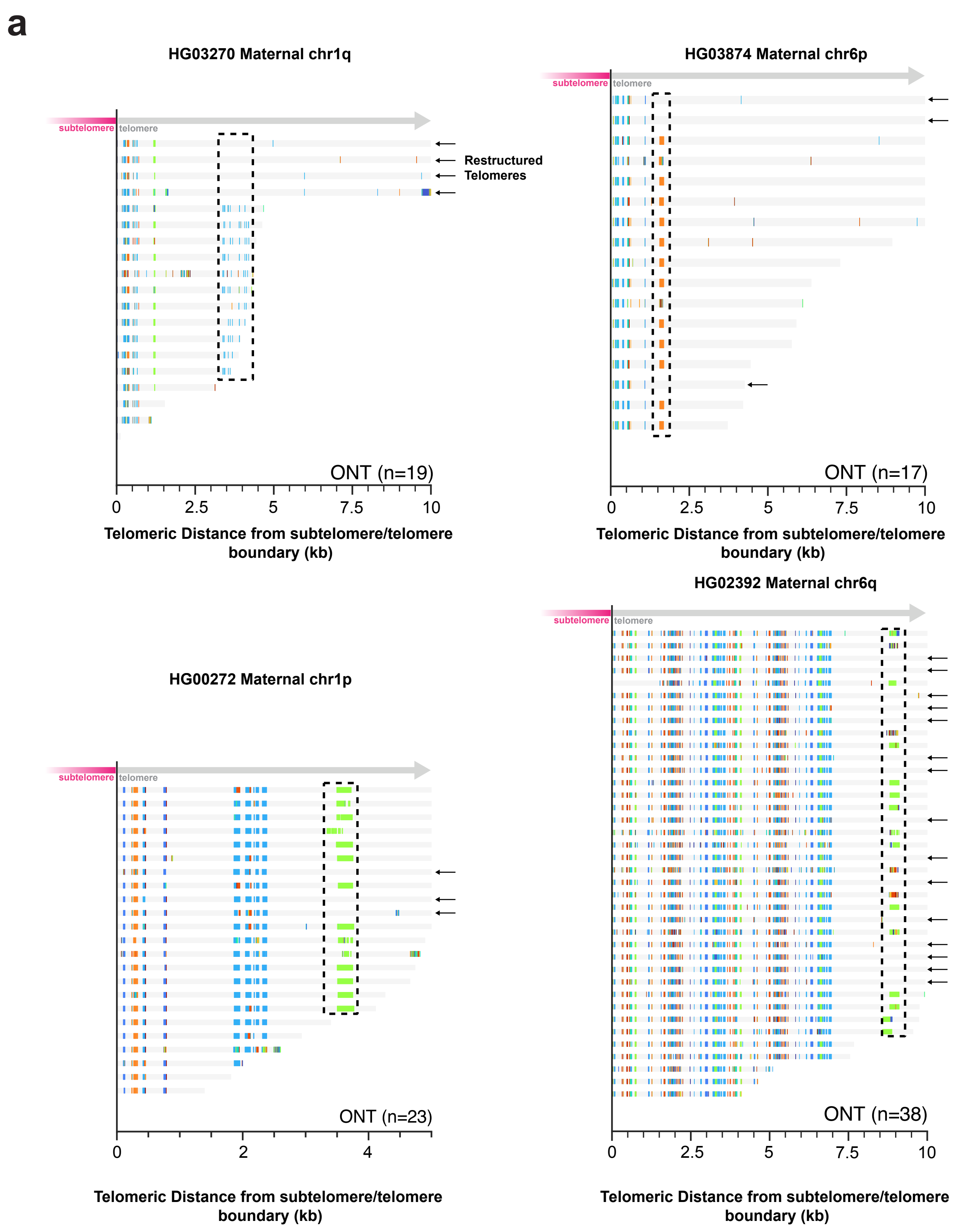

Supplemental Figure 5

a

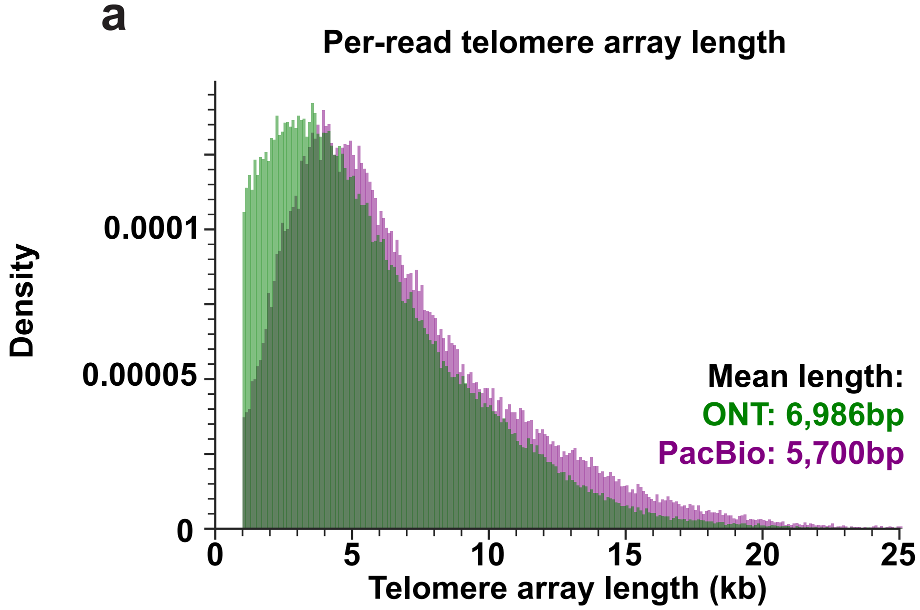

Supplemental Figure 6

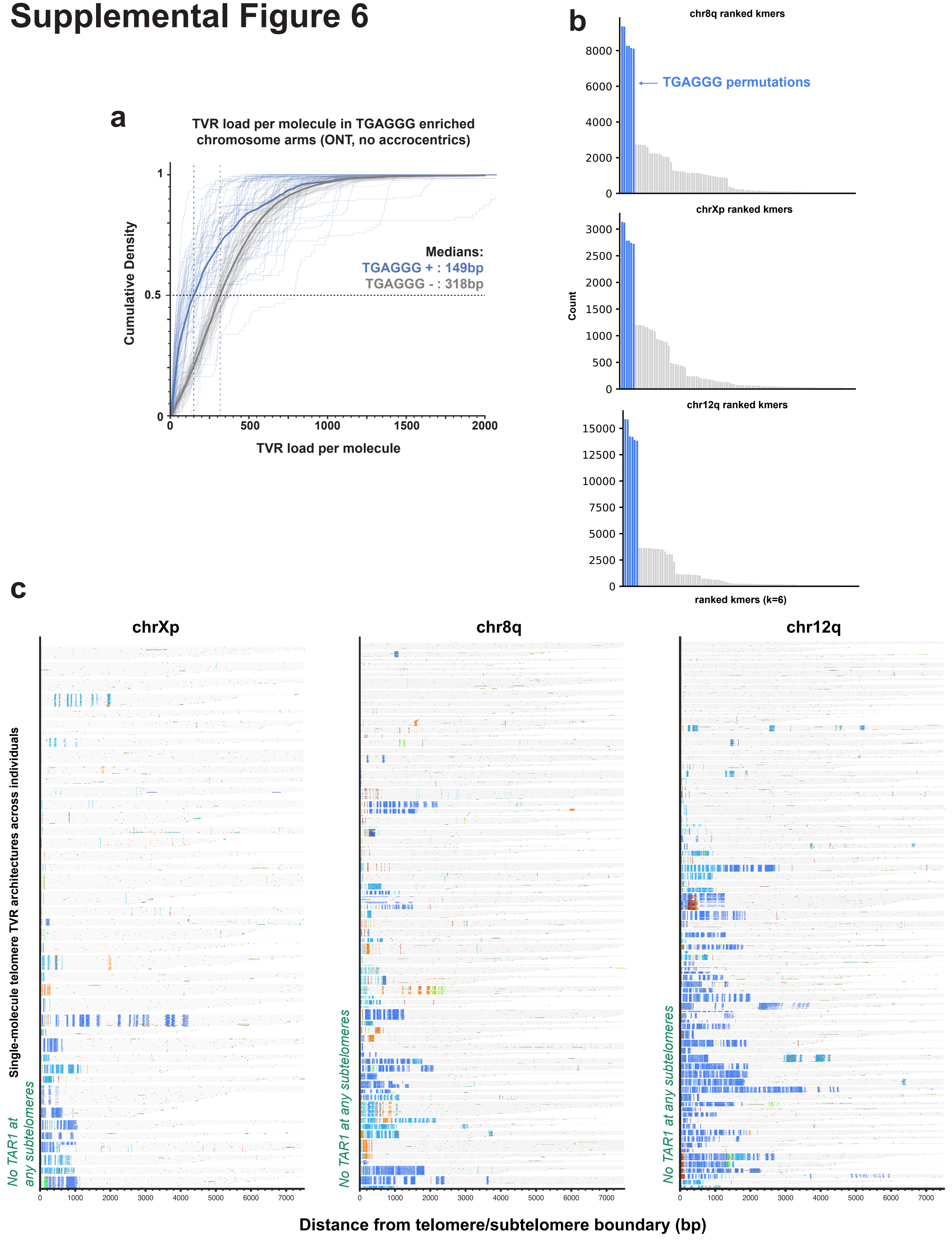

Supplemental Figure 7

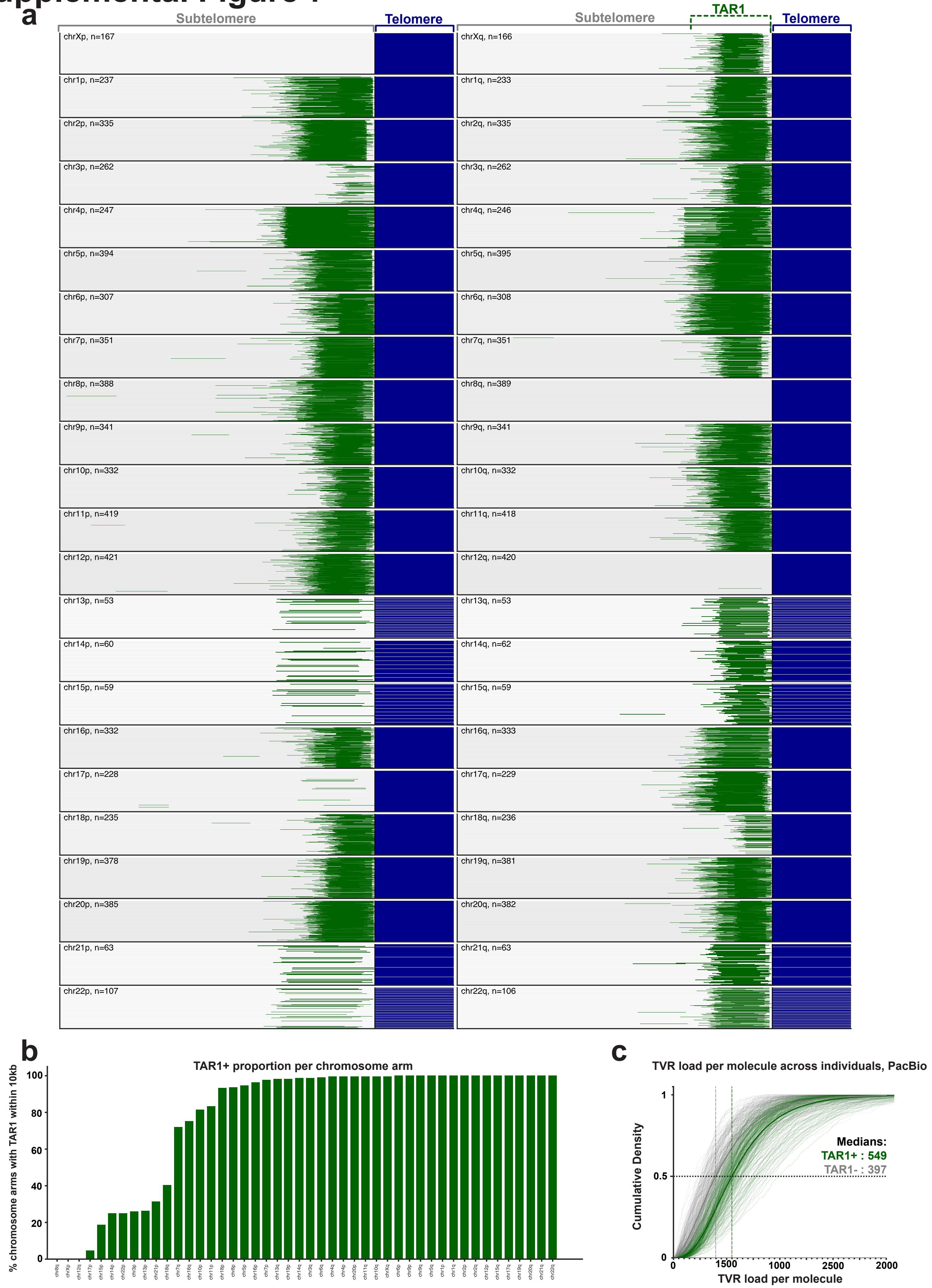

Supplemental Figure 8

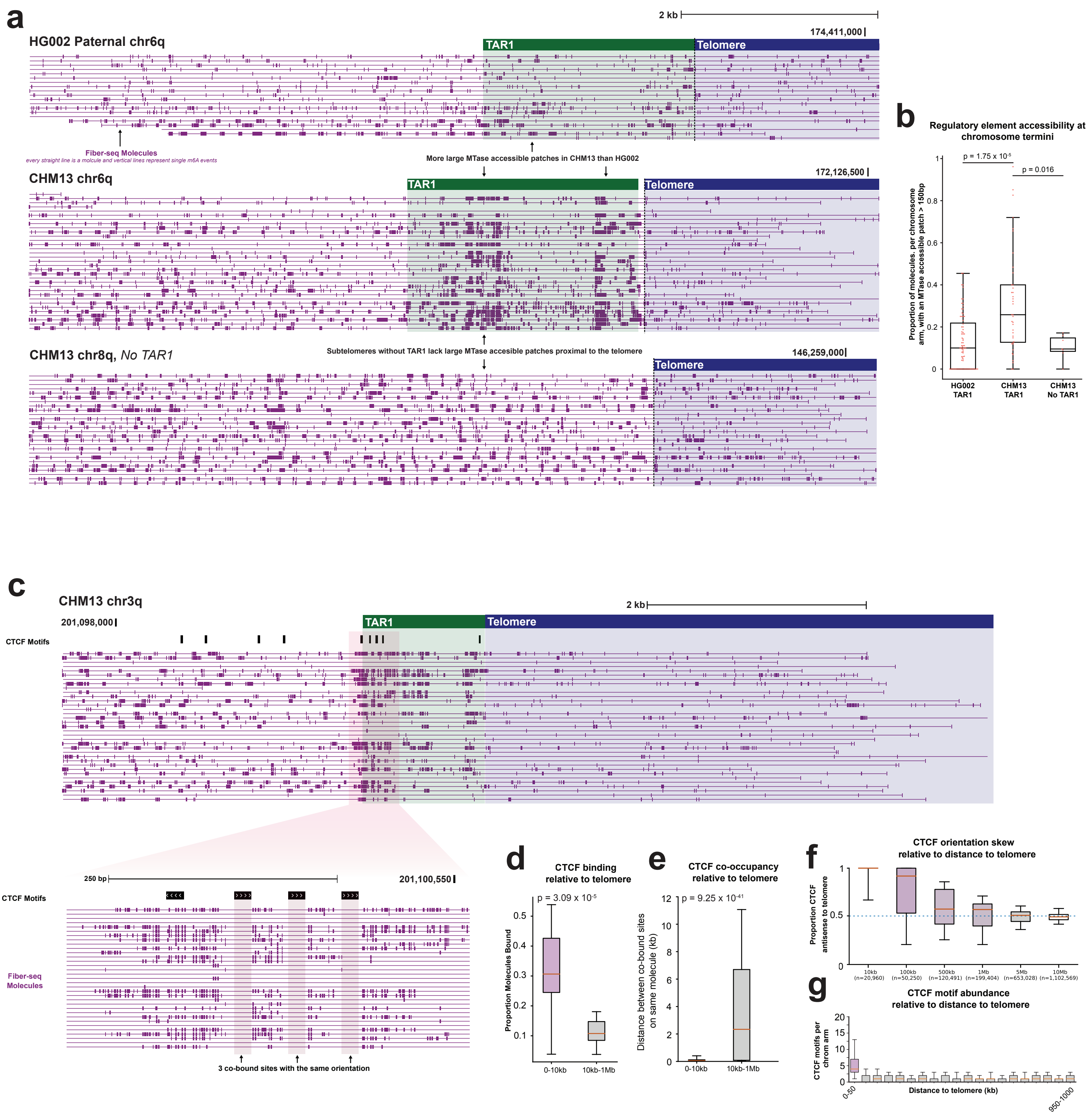

Supplemental Figure 9

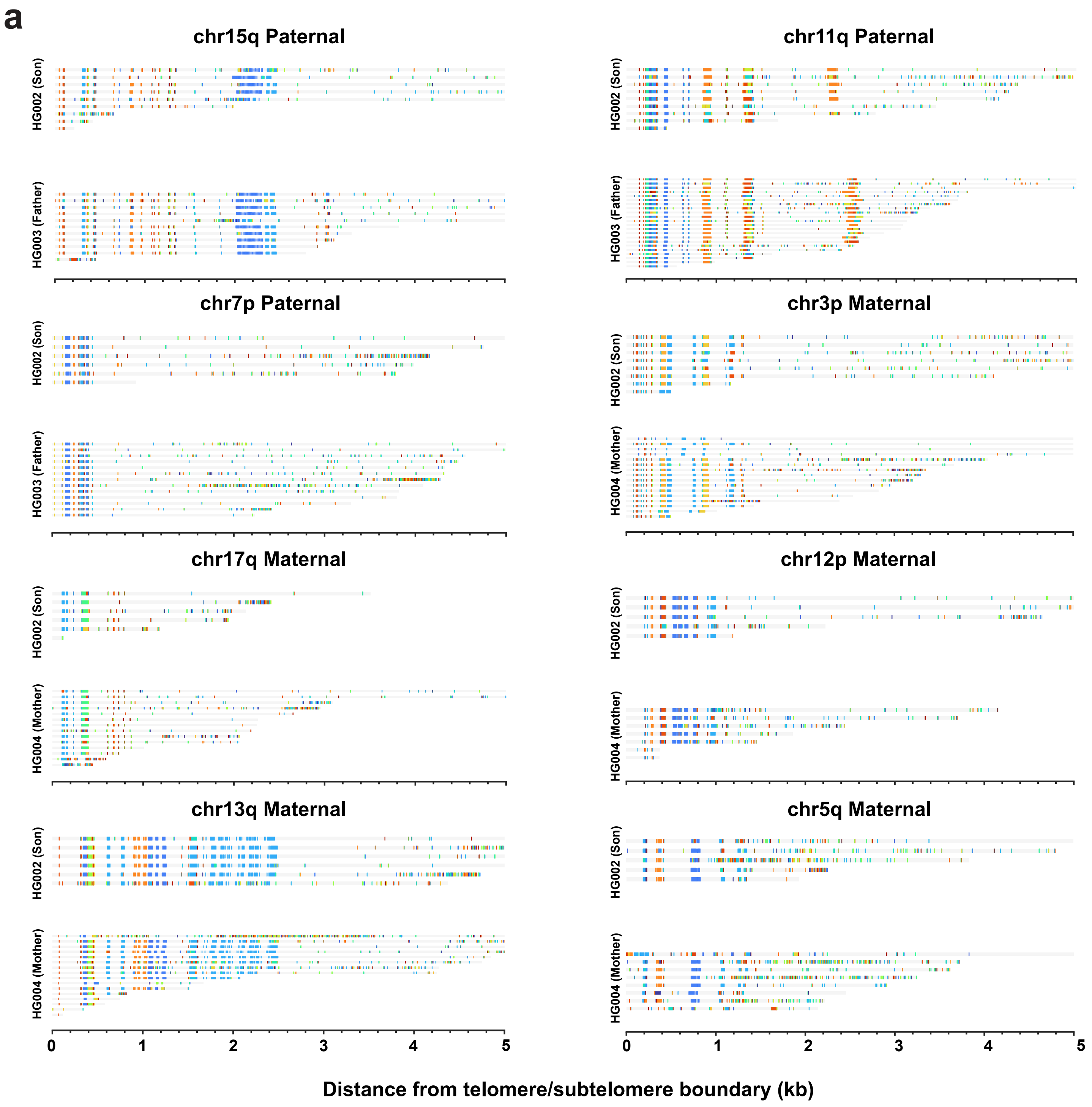

Supplemental Figure 10

a

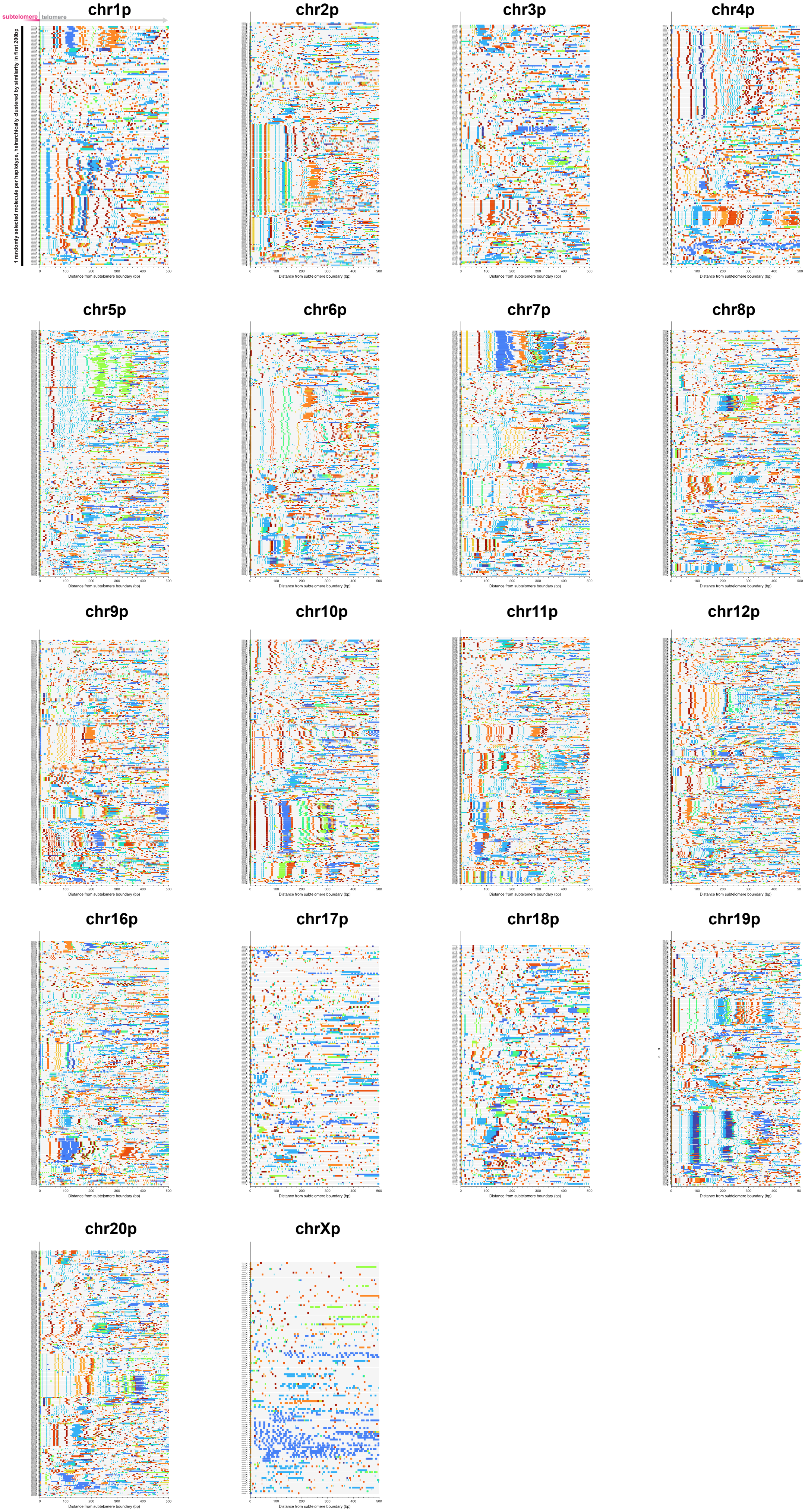

Supplemental Figure 11

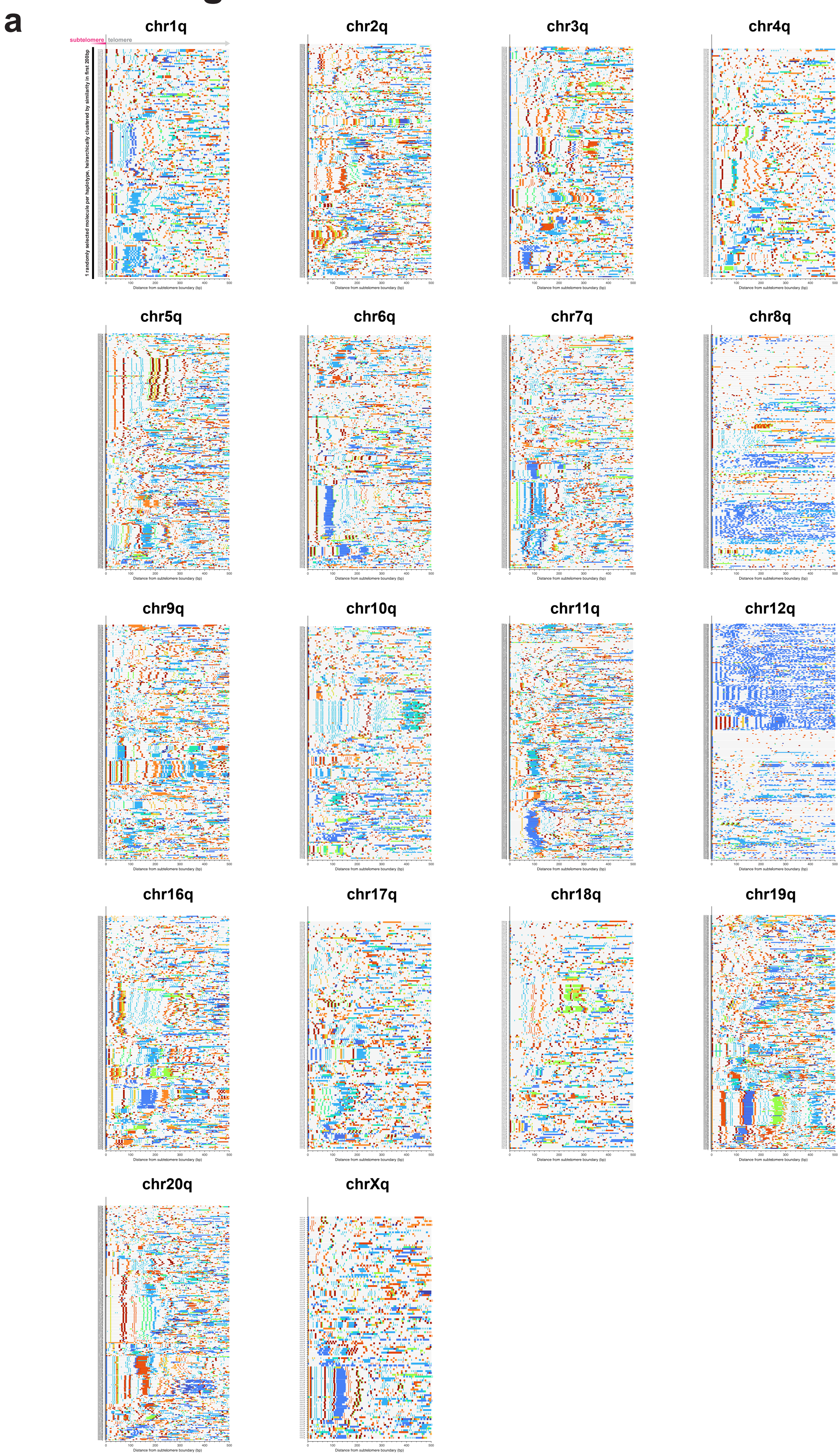

Supplemental Figure 12

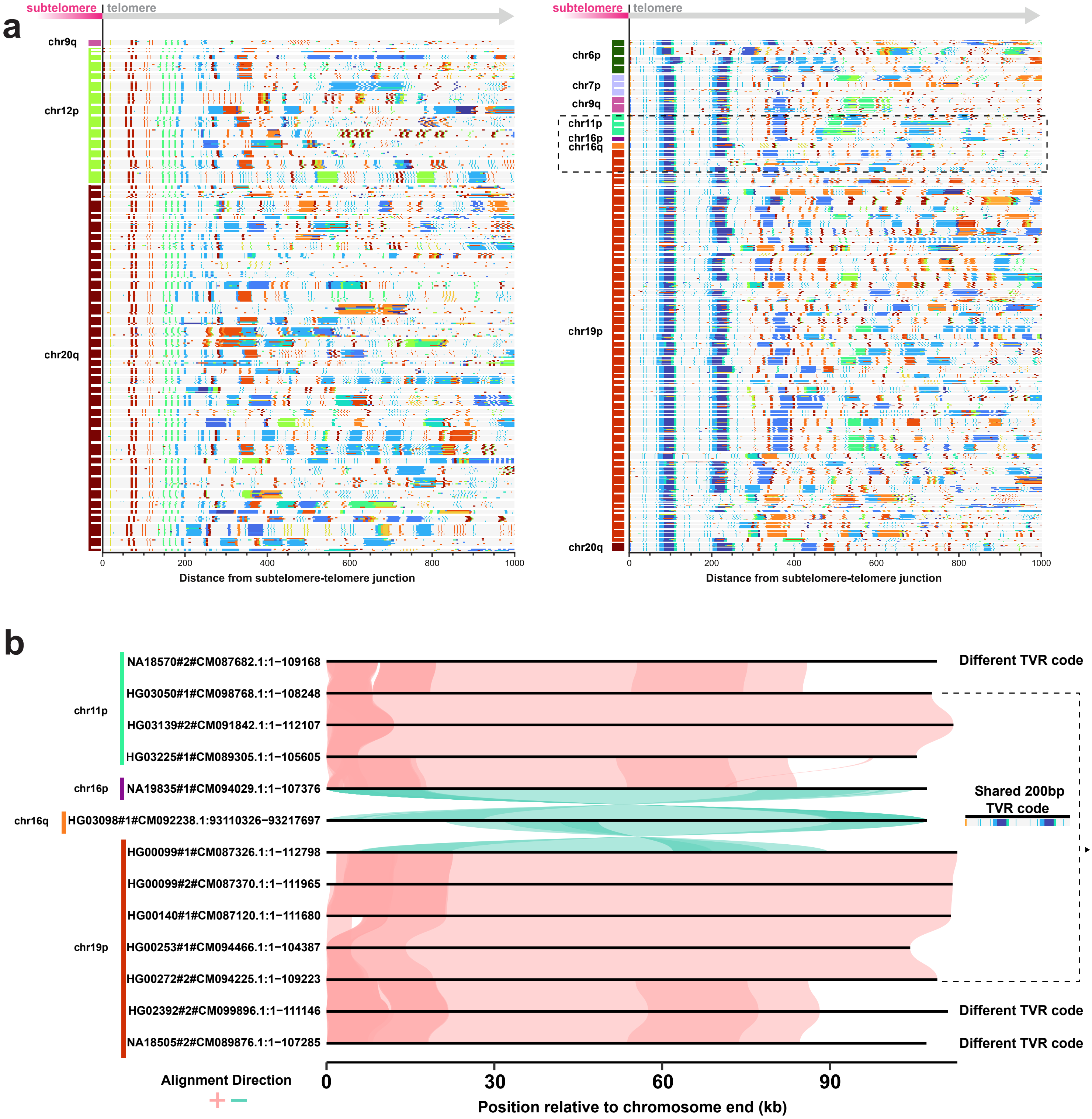

Supplemental Figure 13

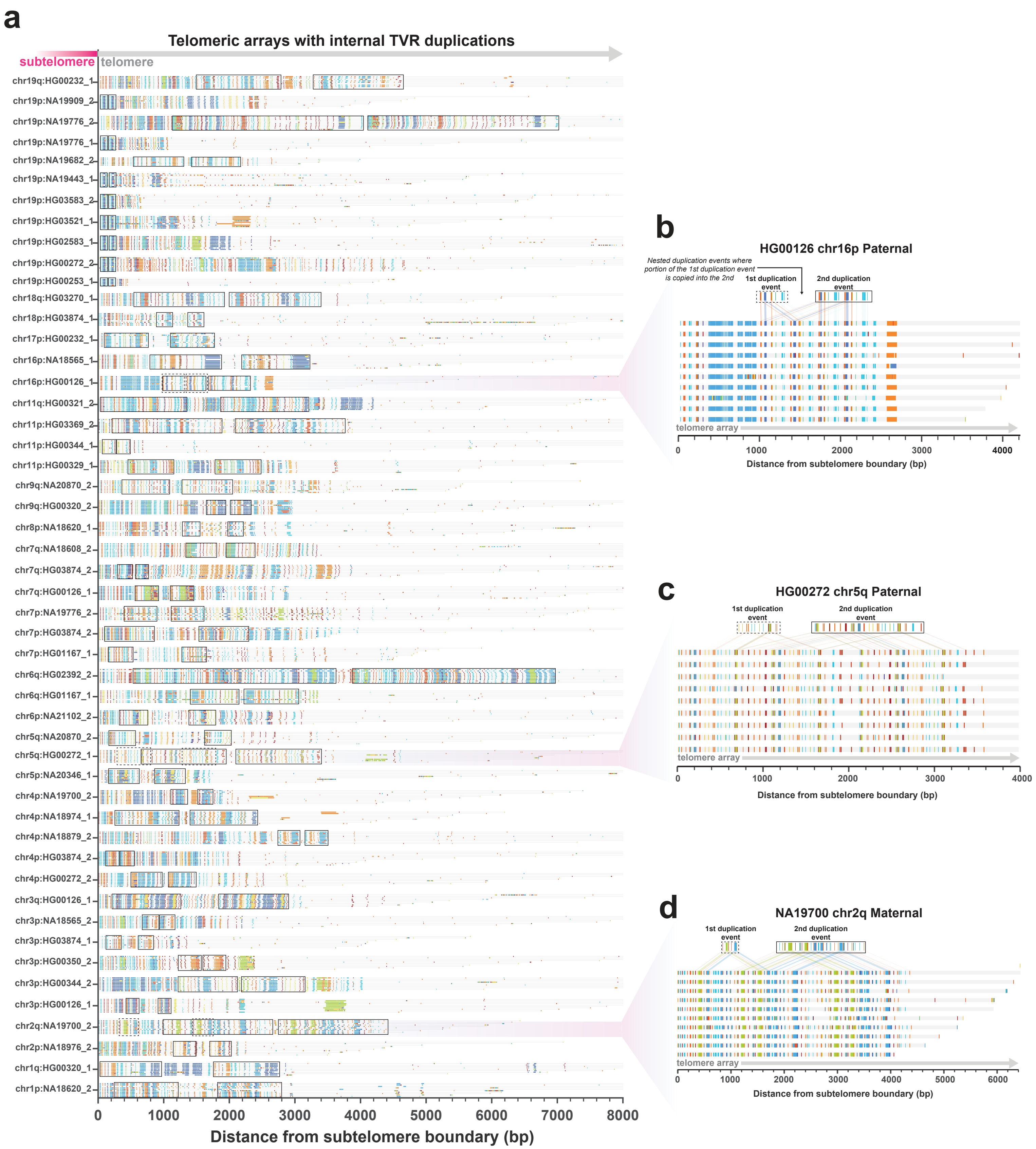

Supplemental Figure 14

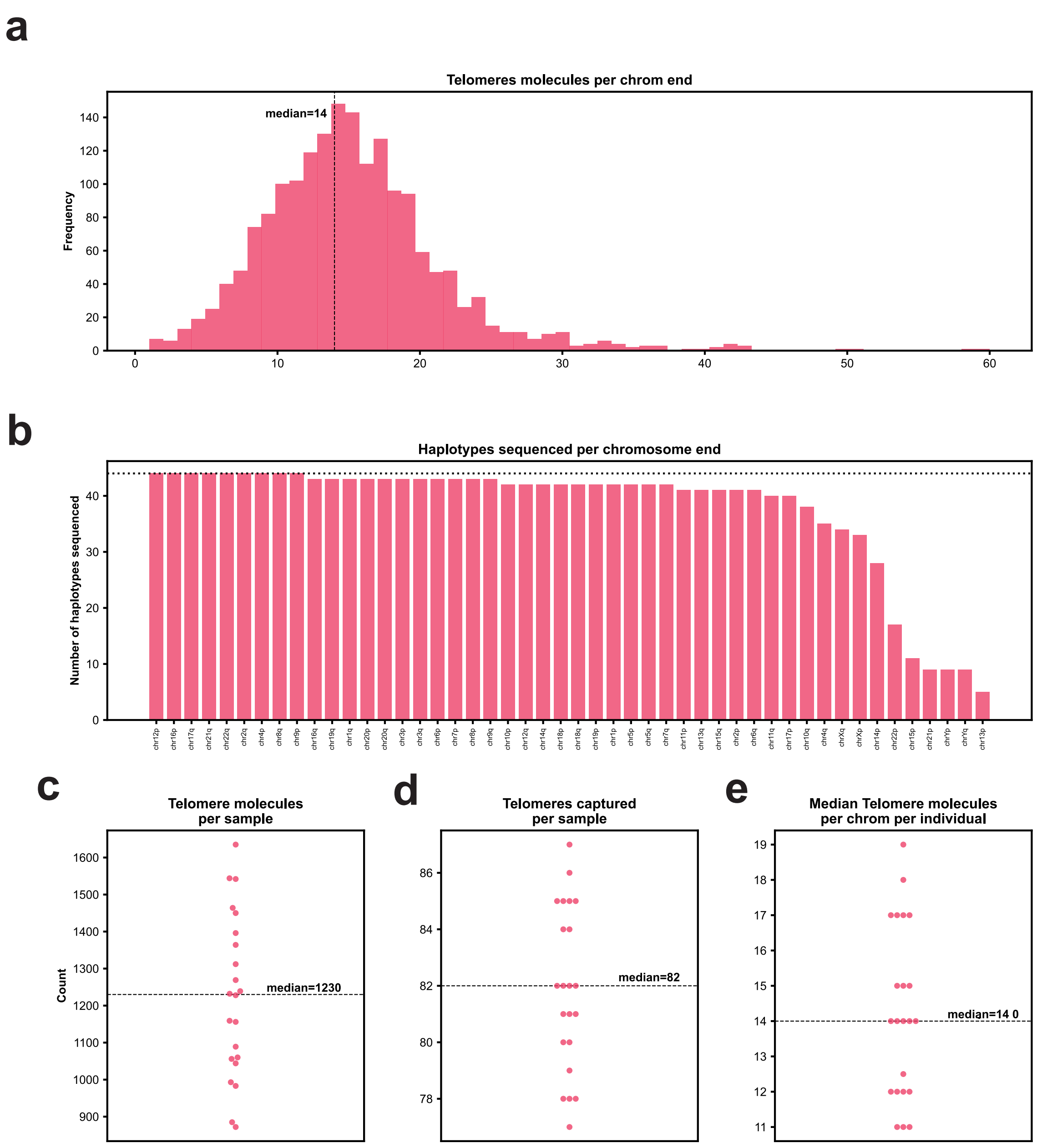

Supplemental Figure 15

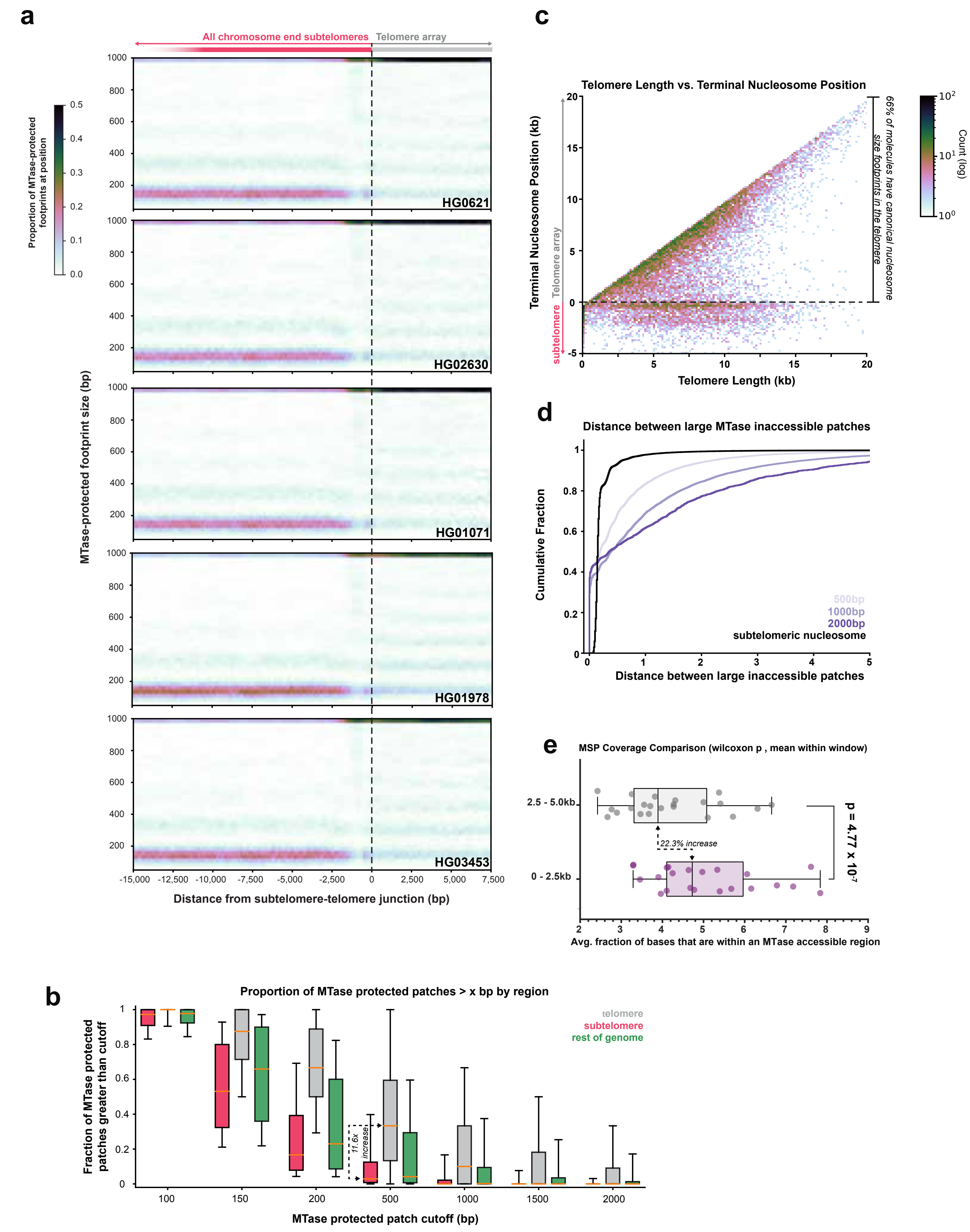

Supplemental Figure S16

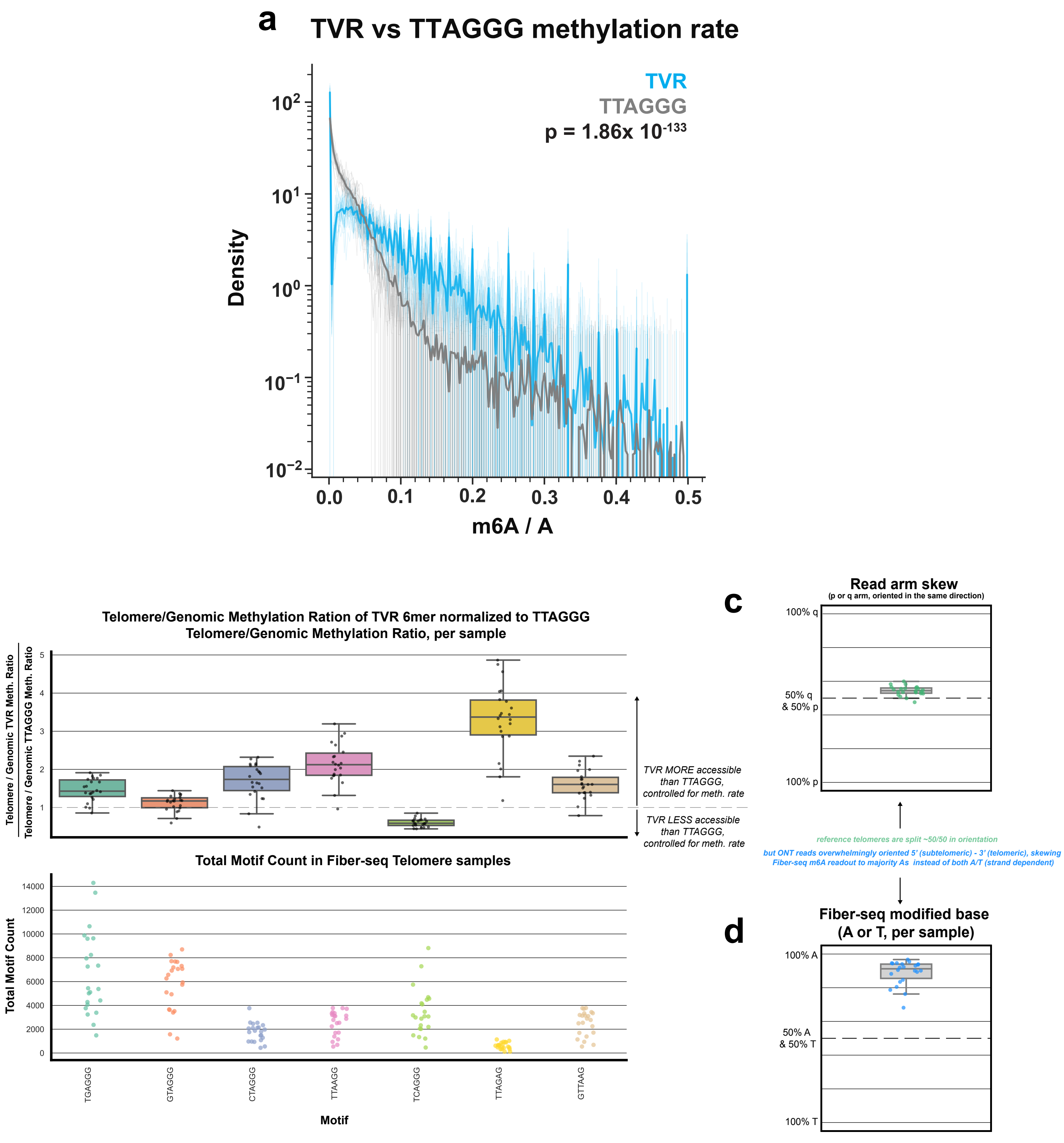

Supplement: Supplement 1 — Fig. S1 | a, PacBio HiFi read alignment depth from HG00099 aligning at the terminal ~100kb of the chromosome in a donor-specific assembly (DSA, top), the CHM13 haploid assembly (middle), and HG002 diploid assembly (bottom) b, Proportion of primary alignments with MAPQ values above or equal to various thresholds in the terminal 100kb of the chromosome arms when aligning to DSA vs HG002 (diploid) and between the sequencing technologies used in this study (PacBio HiFi and ONT R10). c, NM tag value of reads (ONT R10 Fiber-seq reads, aligned to DSA) in the terminal 100kb of the chromosome arms, partitioned into reads overlapping the telomeres and those not overlapping the telomeres. Fig. S2 | a, f, Histogram of the number of telomeric molecules per chromosome end in ONT R10 (a) and PacBio HiFi (f) samples. b, g, Number of haplotypes with an identified terminal telomeric repeat per chromosome arm for ONT R10 (b) and PacBio HiFi (g) datasets. c, h, Total number of telomeric long-read molecules per sample anchored at an identified subtelomere–telomere boundary in ONT R10 (c) and PacBio HiFi (h) datasets, per individual. d, i, Number of chromosome arm termini per sample with at least one molecule anchored at the subtelomere–telomere junction for ONT R10 (d) and PacBio HiFi (i) samples. e, j, Median number of anchored telomeric molecules per chromosome end, per individual, for ONT R10 (e) and PacBio HiFi (j) data. Fig. S3 | a, Comparison of single-molecule TVR calls between ONT R10 and PacBio HiFi data. Single-molecule plots show TVRs ranging from 6 bp to 300 bp in size. b, TVRs across single molecules from all chr5q ends in the HPRC dataset (ONT R10). Each row represents a single read, with gray indicating TTAGGG repeats and color representing specific TVRs according to a shared color map. Data is filtered to include a minimum of 5 reads per individual haplotype end, with TVR sizes ranging from 1 to 300 bp. c, Distribution of TVR block sizes across all PacBio HiFi molecules [file media-1.pdf]
